# Effective detection of human adenovirus in hawaiian waters using enhanced pcr methods

**DOI:** 10.1186/1743-422X-8-57

**Published:** 2011-02-08

**Authors:** Hsin-I Tong, Yuanan Lu

**Affiliations:** 1 Departments of Public Health Sciences and Microbiology, University of Hawaii, Honolulu, 96822, Hawaii

## Abstract

**Background:**

The current criteria for recreational water quality evaluation are primarily based on measurements of fecal indicator bacteria growth. However, these criteria often fail to predict the presence of waterborne human pathogenic viruses. To explore the possibility of direct use of human enteric viruses as improved human fecal contamination indicators, human adenovirus (HAdV) was tested as a model in this study.

**Findings:**

In order to establish a highly sensitive protocol for effective detection of HAdV in aquatic environments, sixteen published PCR primer sets were re-optimized and comparatively evaluated. Primer sets nehex3deg/nehex4deg, ADV-F/ADV-R, and nested PCR primer sets hex1deg/hex2deg and nehex3deg/nehex4deg were identified to be the most sensitive ones, with up to 1,000 fold higher detection sensitivity compared to other published assays. These three PCR protocols were successfully employed to detect HAdV in both treated and untreated urban wastewaters, and also in 6 of 16 recreational water samples collected around the island of Oahu, Hawaii.

**Conclusions:**

Findings from this study support the possible use of enteric viruses for aquatic environmental monitoring, specifically for the essential routine monitoring of Hawaiian beach waters using the optimized PCR protocol to detect HAdV at certain water sites to ensure a safe use of recreational waters.

## Findings

Occurrence of enteric virus contamination in recreational waters has been a major health concern worldwide in recent years [1-5]. However, the current recreational water quality criteria based on the concentration of fecal bacterial indicators (total coliforms, fecal coliforms, and enterococci) often fail to predict the presence of human pathogenic enteric viruses [6-9]. Therefore, enteric viruses have been suggested as alternative indicators of fecal contamination in aquatic environments [6,10,11] due to their low infectious dose [12,13], long survival period in the environment [6,14,15], high resistance to several wastewater treatments [16-18], and the stringent host specificity that makes them free of environmental multiplicity [11]. In addition, detection of human enteric viruses using the library-independent molecular methods (i.e. PCR) is much less laborious and time-consuming compared to the traditional growth-based assessment [11].

The European union regulation has already listed enteroviruses as a parameter governing water quality [11]. However, many studies have suggested that human adenovirus (HAdV), the only enteric virus with double-stranded DNA instead of RNA genome, would make a better candidate as a fecal pollution indicator because of its known stability and persistence in aquatic environments compared to other enteric viruses [11,18,19].

It is well known that monitoring the presence of enteric viruses could be challenging due to the relatively low level of viral particles existing in environmental waters. However, this limitation can be overcome by the use of improved methods for aquatic sample concentration, viral nucleic acids extraction, and more sensitive viral detection techniques [11]. There are currently a number of PCR protocols reported in literature for HAdV detection. However, little is known about their sensitivity or specificity for HAdV detection, particularly for detecting HAdV in environmental waters.

In this study, a total of 16 sets of published HAdV PCR primers, including 3 primer sets for nested PCR, were tested in a side-by-side comparison using a single source of viral DNA for determining detection sensitivity. The selected primer sets were summarized in Table 1. All primer sets were initially tested under standard laboratory PCR conditions with single source HAdV DNA extracted from an *in vitro *cultured cell sample using QIAamp DNA mini kit (Qiagen, CA) according to the manufacturer's instructions. As shown in Table 2, among all 16 sets tested, only 8 primer sets including hex1deg/hex2deg, nehex3deg/negex4deg, ADV-F/ADV-R, XuHex1/XuHex2, hexDEGF/hexDEGR, AdF/AdR, AdV1/AdV2, and AdV3ne/AdV4ne were able to generate PCR products of the expected sizes in a 25 μL volume reaction containing 1X *Taq *reaction buffer (Mg^2+ ^free) (New England Biolabs, NEB, MA), 1.5 mM MgCl_2 _solution (NEB, MA), 200 nM of each dNTP (Sigma-Aldrich, MO), 0.1 μg/μL of BSA (NEB, MA), 400 nM of forward and reverse primers (Integrated DNA technologies, IA), and 2 units of *Taq *DNA polymerase (provided by Dr. Tung Hoang, University of Hawaii at Manoa), with a Master Cycler Gradient (Eppendorf, Germany). Amplification started with an initial denaturation at 94°C for 5 min, followed by 40 cycles of denaturation at 94°C for 30 sec, annealing at 56°C for 30 sec, extension at 72°C for 30 sec, and a final extension at 72°C for 5 min. All PCR products were subjected to 2% agarose gel electrophoreis alongside a 50-bp DNA marker (NEB, MA), stained with Ethidium Bromide (Sigma-Aldrich, MO) and viewed with the Molecular Imager Gel Doc XR+ system (BioRad Laboratories Inc., CA).

**Table 1:** Oligonucleotide sequences used for detection of HAdV

Primer	Sequence (5'→ 3')^a^	+/-^b^	Target	Ampliconsize (bp)	References
Q-Padv-F	AACGGCCGCTACTGCAAG	+	Swine AdV hexon	68	Hundesa *et al*., 2009 [24]
Q-Padv-R	AGCAGCAGGCTCTTGAGG	-			

hex1deg (outer)	GCCSCARTGGKCWTACATGCACATC	+	Hexon	301	Allard *et al*., 2001 [25]
hex2deg (outer)	CAGCACSCCICGRATGTCAAA	-			
nehex3deg (inner)	GCCCGYGCMACIGAIACSTACTTC	+		171	
nehex4deg (inner)	CCYACRGCCAGIGTRWAICGMRCYTTGTA	-			

ADV-F	GCCACGGTGGGGTTTCTAAACTT	+	Hexon	131	Gunson *et al*., 2009 [26]
ADV-R	GCCCCAGTGGTCTTACATGCACATC	-			

XuHex1	TTCCCCATGGCICAYAACAC	+	Hexon	482	Xu *et al*. 2000 [27]
XuHex2	CCCTGGTAKCCRATRTTGTA	-			

hexDEGF	CAGGACGCCTCGGRGTAYCTSAG	+	Hexon	103	Damen *et al*., 2008 [28]
hexDEGR	GGAGCCACVGTGGGRTT	-			

AdE1	TCCCTACGATGCAGACAACG	+	Fiber	967	Xu *et al*. 2000 [27]
AdE2	AGTGCCATCTATGCTATCTCC	-			
AdF1	ACTTAATGCTGACACGGGCAC	+		541-586	
AdF2	TAATGTTTGTGTTACTCCGCTC	-			
AdF	CWTACATGCACATCKCSGG	+	Hexon	~75	Hernroth *et al*., 2002 [29]
AdR	CRCGGGCRAAYTGCACCAG	-			

HAdV-ABCDEF-hexon25f^c^	CARTGGKCDTACATGCACATC	+	Hexon		Kuo *et al*., 2009 [30]
HAdV-E-hexon373r	CCAGRCTGTTGTAGGCAGTG	-		349	
HAdV-F-hexon265r	CCACGGCCAGCGTAAAGC	-		241	

hexAA1885 (outer)	GCCGCAGTGGTCTTACATGCACAGC	+	Hexon	300	Allard *et al*., 1990 [31]
hexAA1913 (outer)	CAGCACGCCGCGGATGTCAAAGT	-			
nehexAA1893 (inner)	GCCACCGAGACGTACTTCAGCCTG	+	Hexon	142	Allard *et al*., 1992 [32]
nehexAA1905 (inner)	TTGTACGAGTACGCGGTATCCTCGCGGTC	-			

JTVFF	AACTTTCTCTCTTAATAGACGCC	+	Fiber	117	Jothikumar *et al*., 2005 [33]
JTVFR	AGGGGGCTAGAAAACAAAA	-			

AdV1 (outer)	CAAGATGGCCACCCCCTCG	+	hexon	329	Oh *et al*., 2003 [34]
AdV2 (outer)	CGATCCAGCACGCCGCGGATGTC	-			
AdV3 (inner)	AATGGTCTTACATGCACAT	+		253	
AdV4 (inner)	ACCCGGTTGTCGCCCACGGCCAG	-			

a. R = A+G; Y = C+T;S = C+G;W = A+T;H = A+C+T;B = C+G+T;V = A+C+G;D = A+G+T;N = all

b. Polarity

c. Forward primer for both HAdV-E-hexon373r and HAdV-F-hexon265r

**Table 2:** Optimized PCR conditions and detection limits for each primer set

Primer	Std^a ^	Optimized condition			Detection limit
			
		T_annel_	[MgCl_2_]	[primer]	
hex1deg/hex2deg	✓	54-60	1.5 mM	600-800 nM	10^-5 ^X
nehex3deg/nehex4deg	✓	58-60	2.0 mM	600-800 nM	10^-6-7 ^X
ADV-F/ADV-R	✓	51.6-55.4	1.5 mM	600-1000 nM	10^-7 ^X
XuHex1/XuHex2	✓	54-60	1.5 mM	1000 nM	10^-5 ^X
hexDEGF/hexDEGR	✓	59.2	3.0 mM	600-800 nM	10^-5 ^X
AdF/AdR	✓	60	1.5 mM	600 nM	10^-6-7 ^X
AdV1/AdV2	✓	51.6-55.4	2.0 mM	600-800 nM	10^-4^X
AdV3ne/AdV4ne	✓	55.4	1.5 mM	400-800 nM	10^-4 ^X
Hex1deg/hex2deg; nehex3deg/nehex4deg^b ^					10^-7 ^X
AdV1/AdV2; AdV3ne/AdV4ne^b ^					10^-5 ^X

a. Standard condition as described in text.

b. Nested PCR.

The 8 primer sets that successfully generated products of respective sizes (Table 1) were subjected to PCR condition optimization. Different annealing temperatures, MgCl_2 _concentration and amounts of primers were combined and adjusted to improve the sensitivity of individual detection protocols. The sensitivity of the detection assay was first evaluated at 6 annealing temperatures ranging from 50°C to 60°C. Using the obtained optimal annealing temperature, each set then was tested at 4 selected MgCl_2 _concentrations ranging from 1.5 to 4.0 mM. The last optimization step was to evaluate the effect of selected primer concentrations from 0.2 μM to 0.8 μM on detection sensitivity. The final optimized PCR conditions for each primer set are listed in Table 2. Detection sensitivity of each primer set was evaluated under the final optimized condition using a serial of 10-fold dilution of a single-source HAdV DNA as template. The detection limits were based on the highest dilution that gave a clear positive signal after PCR amplification. As shown in Table 2, the sensitivities among all tested primers ranged from 10^-4 ^X to 10^-7 ^X dilution, indicating a detection difference of 1,000 fold. Among all, sets nehex3deg/nehex4deg, ADV-F/ADV-R, and AdF/AdR were identified to be the most sensitive ones for ADV detection. Nested PCR using hex1deg/hex2deg and nehex3deg/nehex4deg exhibited similar sensitivity by showing the positive results with stronger detection signals when the same dilution was used. Due to the small size of the final product generated by AdF/AdR (less than 100 bp), only nehex3deg/nehex4deg, ADV-F/ADV-R, and nested PCR sets hex1deg/hex2deg and nehex3deg/nehex4deg were employed in a survey study directed to testing sewage and environmental water samples for naturally occurring HAdV and the detection protocol validation.

**Figure 1:**
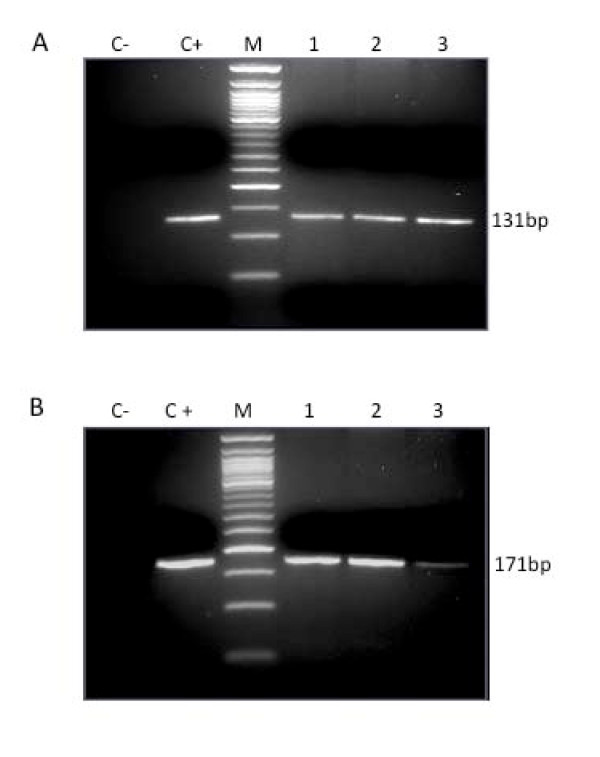
**Agarose gel electrophoresis of PCR detection of HAdV in urban wastewaters of three different treatments stages**. (A) Amplified with primer set ADV-F/ADV-R. (B) amplified with primer set nehex3deg/nehex4deg. HAdV were detected from 100 mL of untreated raw influenced (lane 1), pre-disinfection (lane 2), and post-disinfection/effluence (lane 3) stages. Lane M = 50-bp DNA marker, lane C+ = positive control using HAdV DNA, and lane C- = no template control.

From previous experience examining human norovirus contamination, we learned that PCR conditions optimized using a single clinical source of viral nucleic acids might not act as specifically on total nucleic acids extracted from a complex microbial community, especially when the target viruses only exist in a very small amount compared to other organisms, which is often the case for naturally occurring enteric viruses in environmental waters. Urban wastewaters of human origin are known to contain a large volume of enteric viruses due to characteristic shedding in high numbers in the feces of infected individuals [11,20]. Therefore, the three selected protocols were first validated for their potential application in HAdV detection in environmental samples by testing treated and untreated urban wastewater samples collected from Sand Island Wastewater Treatment Plant (SIWWTP, Hawaii) during May 2010. The SIWWTP processes around 60 million gallons of wastewater daily, accounting for approximately 85% of Oahu's wastewater. Samples were collected from 3 different treatment stages, including untreated raw influence, post-primary clarifying/pre-disinfection stage, and post-disinfection/effluence. Water samples were concentrated according to the filtration-based method described by Katayama *et al*. [21] with modifications. Briefly, negatively charged type HA filter membranes (Millipore Corporation, MA) with a 0.45-μm pore size and 90-mm diameter were used with a vacuum pump system. MgCl_2 _was added to the sewage samples at a final concentration of 25 mM before filtration was performed. One hundred milliliters of wastewater sample was filtered through the membranes for viral absorption. The recovered membranes were subjected to nucleic acid extraction using the PowerWater DNA isolation kit (MoBio Laboratories, CA) according to the manufacturer's instructions. Five microliters of the total DNA was used as the template for HAdV detection using the optimized PCR protocols with primer sets ADV-F/ADV-R and nehex3deg/nehex4deg. As expected, HAdV was detected consistently by the two detection methods in all three stages of the collected urban wastewater samples with clean, single bands as shown in Figure 1.

Following validation with sewage samples, we evaluated the three protocols for potential use in detecting naturally occurring HAdV in environmental water samples. Sixteen surface water samples were collected from different recreational water bodies composed of both marine and fresh waters around the island of Oahu during June 2010. Sample sites, conditions, and filtration volumes are summarized in Table 3. To ensure the reliability of water filtration and nucleic acid extraction processes, a field blank sample comprised of 2 L distilled water as a negative control, and a spiked sample made by adding 50 mL of HAdV-positive untreated wastewater sample into 2 L of seawater collected from Diamond Head Beach Park as a positive control were also carried out using the same filtration and DNA extraction procedures for all environmental samples. Surface waters collected from fresh water bodies were subjected to initial treatment with 25 mM MgCl_2 _for 5 min at room temperature prior to filtration.

**Table 3:** HAdV prevalence in Hawaiian urban wastewater and environmental waters

Sample			PCR		Nested PCR	
					primary	nested
**Site**	**Condition**	**Volume**	**ADV-F ADV-R**	**nehex3deg nehex4deg**	**hex1deg hex2deg**	**hex1deg/hex2deg; nehex3deg/nehex4deg**

SIWWTP influence tank	Sewage	100 mL	**+**	**+**	**+**	ND

SIWWTP clarifying tank	Sewage	100 mL	**+**	**+**	**+**	ND

SIWWTP effluence tank	Sewage	100 mL	**+**	**+**	**+**	ND

Sand Island State Recreational Area	Seawater	2.00 L	**-**	**-**	**-**	**+**

Kailua Bay	Seawater	2.00 L	**+**	**-**	**-**	**+**

Waikiki Beach	Seawater	2.00 L	**-**	**+**	**-**	**+**

Ala Wai Canal	Freshwater	2.00 L	**-**	**-**	**-**	**+**

Wahiawa freshwater	Freshwater	2.00 L	**-**	**-**	**-**	**+**

Manoa stream	Freshwater	2.00 L	**+**	**+**	**-**	**+**

Ala Moan Park/Magic Island	Seawater	2.00 L	**-**	**-**	**-**	**-**

Diamond Head Beach Park	Seawater	2.00 L	**-**	**-**	**-**	**-**

Maili Beach Park	Seawater	2.00 L	**-**	**-**	**-**	**-**

Waianae/Pokai Bay	Seawater	2.00 L	**-**	**-**	**-**	**-**

Waialae Beach Park	Seawater	2.00 L	**-**	**-**	**-**	**-**

Maunalua Bay Beach Park	Seawater	2.00 L	**-**	**-**	**-**	**-**

Kalaka Bay Beach Park	Seawater	2.00 L	**-**	**-**	**-**	**-**

Bellows Field Beach Park	Seawater	0.80 L^a^	**-**	**-**	**-**	**-**

West Loch Community Shoreline Park	Seawater	0.80 L^a^	**-**	**-**	**-**	**-**

Kailua Stream	Freshwater	0.50 L^a^	**-**	**-**	**-**	**-**

Field Blank	dH_2_O	2.00 L	**-**	**-**	**-**	**-**

Spike control	S+S^b^	2 +0.05 L	**+**	**+**	**+**	**+**

a. 2L sample water was initially prepared but actual volume passed through the filter membrane.

b. 2 L of seawater + 50 mL of sewage water.

c. ND = not done

As shown in Table 3, six sites including Sand Island State Recreational Area, Kailua Bay, Waikiki Beach, Ala Wai Canal, Wahiawa freshwater, and Manoa stream were all positive for HAdV by using the nested PCR protocol with hex1deg/hex2deg and nehex3deg/nehex4deg. As a comparison, Kailua Bay and Manoa stream were HAdV positive using the ADV-F/ADV-R PCR protocol, while HAdV was found in the surface waters of Waikiki Beach and Manoa Stream when nehex3deg/nehex4deg PCR protocol was employed. Manoa Stream was the only site at which all three protocols confirmed HAdV presence. All HAdV positive PCR products were recovered after electrophoresis from 2% agarose gel using QIAquick Gel Extraction kit (Qiagen, CA) by following the manufacture's instruction. The recovered DNAs were sent to the Advanced Studies in Genomics, Proteomics and Bioinformatics (ASGPB, University of Hawaii at Manoa) for sequencing to confirm the positive detection of HAdV.

The resulting positive detection of HAdV from environmental water by employing highly sensitive detection methods clearly indicates that low concentration is not an impossible obstacle to overcome when detecting human enteric viruses from environmental waters. In this study, the comparative analysis of several PCR assays currently available for HAdV detection has led to the identification of three highly sensitive PCR protocols, which were successfully employed for effective detection of HAdV in different types of aquatic environments in Hawaii. In addition, the nested PCR appeared to be superior to the other two protocols for detecting HAdV in environmental waters, suggesting this protocol should be a priority for use in facilitating early detection of HAdV contamination in future.

As the only enteric virus containing a double-stranded DNA genome, HAdV has been shown to be up to 60 times more resistant to UV irradiation than its RNA enteric virus counterparts [17,22] and is able to persist and remain infectious in the environment for a long period of time [6]. In addition, HAdV occurrence was reported to have great correlation with other human enteric viruses, especially hepatitis A virus [19,23]. With several well-established stable cell lines available, HAdV could also be subjected to infectious studies and future exposure risk assessment. Overall, HAdV would make an ideal candidate as a potential molecular index for enteric viral contamination of recreational waters.

It should be noted that this is the first report of HAdV detection in Hawaiian environmental waters using PCR methods to the best of our knowledge. The high prevalence of HAdV in Oahu waters revealed from this study should raise public awareness of a more serious beach contamination issue than previously expected. These new findings strongly argue the importance and necessity of including these established, sensitive HAdV detection protocols into routine water quality monitoring for better protection of the public from recreational waterborne illness associated with enteric viruses. Current research in this laboratory is directed to the establishment of *in vitro *infectivity assays for water samples collected from the HAdV positive sites and to the determination of a possible correlation between the PCR detection and actual viral infectivity, thus providing baseline information important for the interpretation and assessment of PCR-based HAdV detection and actual health risk to the public.

## Competing interests

The authors declare that they have no competing interests.

## Authors' contributions

HT carried out all experimental testing, data analysis, and drafted the manuscript; and YL conceived of the study, experimental design and data analysis, and manuscript revision. Both authors read and approved the final manuscript
